# Evaluation and Validation of a Real-Time Polymerase Chain Reaction Assay for Rapid Identification of Bacillus anthracis

**DOI:** 10.3201/eid0810.020393

**Published:** 2002-10

**Authors:** Alex R. Hoffmaster, Richard F. Meyer, Michael P. Bowen, Chung K. Marston, Robbin S. Weyant, Kathy Thurman, Sharon L. Messenger, Erin E. Minor, Jonas M. Winchell, Max V. Rasmussen, Bruce R. Newton, J. Todd Parker, William E. Morrill, Nancy McKinney, Gwen A. Barnett, James J. Sejvar, John A. Jernigan, Bradley A. Perkins, Tanja Popovic

**Affiliations:** Centers for Disease Control and Prevention, Atlanta, Georgia, USA

**To the Editor:** During the 2001 anthrax outbreak, we evaluated and validated a highly sensitive and specific three-target (two plasmid and one chromosomally located target) 5´ nuclease assay (real-time polymerase chain reaction [PCR]) for detection and identification of Bacillus anthracis. This PCR assay was successfully used to rapidly test hundreds of suspect isolates as well as screen environmental samples for the presence of B. anthracis throughout the 2001 anthrax outbreak. For the first time in an outbreak setting, a PCR assay was used to detect B. anthracis directly from clinical specimens, consequently becoming a part of the laboratory confirmation of anthrax. In this letter, we describe the evaluation of this assay on a diverse panel of bacterial isolates including isolates obtained throughout the outbreak. A supplement, which includes data on the use of this assay on environmental and clinical specimens, is online (available from http://www.cdc.gov/ncidod/EID/vol8no10/02-0393sup.htm).

Identification of B. anthracis has traditionally been determined by using phenotypic differences between B. anthracis and the rest of the B. cereus group (i.e., lack of motility and hemolysis, susceptibility to penicillin, typical colony morphology, and susceptibility to lysis by gamma phage); however, these methods are slow and require at least 24 h for completion. The recent bioterrorism-associated outbreak and the ongoing threat emphasize the importance of rapid microbiologic diagnosis for the timely and adequate implementation of control and preventative measures.

For B. anthracis, the main targets for development of such assays, primarily PCR-based, have been and continue to be genes encoding its virulence factors: a tripartite exotoxin and an antiphagocytic capsule (1–4). The toxin genes (pagA, lef, and cya) are encoded on the 182-kb virulence plasmid, pXO1, while the genes required for capsule biosynthesis (capB, capC, and capA) are encoded on the 96-kb virulence plasmid, pXO2 (5–7). These plasmid-located virulence genes seem to be restricted to B. anthracis, giving the plasmid-based assays a high degree of specificity (8). However, strains of B. anthracis that lack these plasmids have been reported (4,9). Consequently, having an assay focus on a specific chromosomal target for detection of avirulent and plasmid-cured B. anthracis, as well as those that potentially could have been genetically engineered, is essential. Chromosomal markers, such as vrrA and Ba813, have been used to characterize B. anthracis (9–12) and to detect it in tissues of victims of the anthrax outbreak that occurred in 1979 in Sverdlovsk, former Soviet Union (12), but these markers are not restricted to B. anthracis. Recently, Qi et al. developed a fluorescence resonance energy transfer PCR assay that targets the B. anthracis chromosomally located rpoB gene. This assay appears to be the most specific described to date with only 1 of 175 non-B. anthracis bacilli reported as positive (13).

Over the past several years, activities in the area of bioterrorism preparedness in the United States have resulted in the establishment of an international Laboratory Response Network (LRN), which was instrumental in the identification of the agent used in the 2001 outbreak (14). One of the major initiatives of LRN has been development and validation of rapid and specific assays for identification of B. anthracis and other agents likely to be used in a bioterrorism event.

Primer and probe set BA1 targets a region of pX02, BA2 targets pX01, and BA3 targets a region of the B. anthracis chromosome. Probes were labeled with 6-carboxy-fluorescein phosphoramidite and 5-carboxy-tetramethyl-rhodamine.

LRN PCR assays using the BA1, BA2, and BA3 primer and probe sets were performed with the LightCycler (Roche Diagnostics GmbH, Mannheim, Germany), Smart Cycler (Cepheid, Sunnyvale, CA), or ABI Prism 7700 (Applied Biosystems, Foster City, CA) instruments. The LightCycler Faststart DNA master hybridization probes kit (Roche Diagnostics GmbH) reagents were used on all real-time platforms. Reactions comprised 1X reaction mix, 5 mM MgCl2, 500 nM each primer, and 100 nM probe in a reaction volume of 20 µL (LightCycler) or 25 µL (Smart Cycler, ABI Prism 7700). Thermal cycler conditions consisted of an initial 10-min hold at 95°C followed by 40–45 cycles of 10 s (LightCycler) or 15 s (Smart Cycler, ABI Prism 7700) at 95°C and 30 s (LightCycler, Smart Cycler) or 60 s (ABI Prism 7700) at 60°C. Real-time data were collected during the 60°C extension step of each cycle. Amplification of the human βbeta-actin gene was used as a real-time PCR control when used on clinical samples to ensure negative results were not from inhibition of the PCR reaction. This real-time PCR assay was considered positive when all three targets were positive (Figure).

**Figure:**
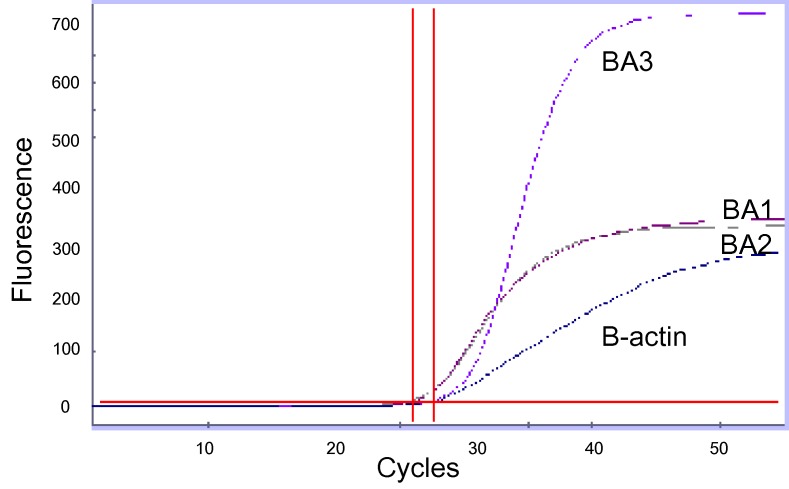
Real-time polymerase chain reaction graph of three B. anthracis markers and B-actin control detected in a pleural fluid specimen from a patient with inhalational anthrax. The horizontal line indicates a threshold value; the vertical lines indicate cross-threshold values for each marker. BA1, primer and probe set targeting a region of pXO2; BA2, primer and probe set targeting a region of pXO1; BA3, primer/probe set targeting a region of B. anthracis chromosome.

A total of 542 isolates were tested. Eighty-one B. anthracis isolates were tested to evaluate sensitivity of the real-time PCR approach (Table). Seventy-five were selected to provide a test population representing diverse sources, genotypes, geographic origins, and dates of isolation. The isolates included those collected from animals, humans, and other sources (i.e., industrial sites associated with anthrax outbreaks); the isolates span at least 58 years (1939–1997). Fifty-three of the isolates were previously characterized by multiple-locus variable-number tandem repeat analysis (MLVA) (15) and were included to ensure a representative range of the 89 described MLVA genotypes to date. Six B. anthracis type and standard strains included: five pXO1 cured strains (including the Pasteur strain) and one pXO2 cured strain (the veterinary vaccine strain Sterne). The B. anthracis New Hampshire strain (16) was used as a positive control for all real-time PCR assays. This isolate was originally cultured from a patient with inhalational anthrax in New Hampshire in 1957. This real-time PCR is designed to identify fully virulent (wild-type) B. anthracis, which will give positive results in all three markers. However, naturally occurring isolates have been found lacking either virulence plasmid, and a number of laboratory strains have been plasmid cured, as well. PCR results for these strains will reflect the lack of one or both of their plasmids.

**Table:** Origin, designations, and results of real-time polymerase chain reaction assay for *Bacillus* *anthracis* strains^a^

				No. positive/total		
*B. anthracis*	No. analyzed	Temporal range and geographic origin	MLVA genotypes represented^a^	Ba1^b^	Ba2^b^	Ba3^b^
Human isolates	30	1943–1996 Africa, Asia, Australia, Europe, North America	3, 4, 22, 23, 28, 32, 34, 35, 36, 37, 41, 43, 44, 45, 50, 66, 68	30/30	30/30	30/30
Animal isolates	29	Africa, Asia, Australia, Europe, North America, South America	3, 10, 20, 26, 29, 30, 35, 38, 40, 45, 48, 49, 51, 55, 57, 78, 80, 81, 84, 85, 87, 89	29/29	29/29	29/29
Other isolates	16	1950–1993 Africa, Asia, Europe, N. America	13, 14, 21, 24, 47, 62, 69, 73, 77, 79, 82	16/16	16/16	16/16
Outbreak isolates	317	2001 U.S. outbreak	62	317/317	317/317	317/317
pXO1 cured	5	1956–1974 North America		5/5	0/5	5/5
pXO2 cured	1	Africa		0/1	1/1	1/1

^a^MLVA, multiple-locus variable-number tandem repeat analysis as described by Keim et al. (15). ^b^Ba1, Ba2, and Ba3 primer/probe sets as described in Materials and Methods.

A total of 317 B. anthracis isolates obtained during the bioterrorism-associated anthrax outbreak from October to December 2001 were also analyzed by PCR. These included 27 isolates from clinical specimens, 4 from powders and 286 isolates from environmental samples. MLVA was performed on 135 of these isolates; all were indistinguishable (17).

For evaluation of the assays’ specificity we tested 56 archived members of the Bacillus genus: B. subtilis (9 strains, 5 clinical, 4 unknown), B. cereus (23 strains, 9 clinical, 14 environmental), B. thuringiensis (12 strains, 6 clinical, 3 insects, 3 unknown), B. mycoides (1 strain, unknown), B. megaterium (10 strains, 7 clinical, 3 unknown), and the environmental Bacillus spp. isolate, Ba813_11, which resulted in a previously reported false-positive result in the B. anthracis-specific PCR assay targeting rpoB (13). In addition, 88 isolates from environmental and clinical specimens, which were confirmed not to be B. anthracis by standard microbiologic methods were tested. These isolates were selected because of their lack of hemolysis and because they had a colony morphology similar to B. anthracis on blood agar plates.

Before testing, all strains were stored at –70°C in brain heart infusion broth (BHIB, Centers for Disease Control and Prevention [CDC], Atlanta, GA) or water containing 20% glycerol. Identification of all strains was confirmed by using standard microbiologic procedures and the LRN testing algorithm (14,18). Colony-lysis DNA preparations were used for all Bacillus spp. strains. Isolates were streaked onto trypticase soy agar containing 5% sheep blood (Becton Dickinson Microbiology Systems, Cockeysville, MD) and incubated overnight at 37°C. A single colony was transferred and dispersed into 0.22 mM centrifugal filter units (Millipore, Bedford, MA) containing 200 mL 10 mM Tris-HCl (pH 8.0). The suspension was heated at 95°C for 20 min and then cooled to room temperature. The filter units were then centrifuged at 6,000 x g in a microfuge for 2 min and the filter discarded. The resulting lysate was stored at –20°C until use.

The lower limit of detection of each assay was tested by using five B. anthracis strains: Ames (2000031656), Pakistan-sheep (2000031648), French-bovine (2000031651), Sterne (2000031075), and Pasteur (2000031759). DNA was extracted from vegetative cells by first pre-treating cell pellets with lysozyme and lysostaphin and then using the MasterPure DNA Purification kit (Epicentre, Madison, WI), following the manufacturer’s protocol for cell samples. B. anthracis spores were quantitated microscopically and tested directly in the real-time PCR assay without DNA extraction. Vegetative-cell DNA was tested at concentrations ranging from 10 ng to 400 fg DNA per reaction. Spores were tested at concentrations ranging from 100,000 spores to 1 spore per reaction. All reactions were performed in duplicate on the LightCycler, Smart Cycler, and ABI Prism 7700 instruments.

All 75 wild-type (fully virulent) B. anthracis isolates tested were positive for all three targets resulting in 100% sensitivity (95% confidence interval [CI] 95% to 100%). Strains cured of pXO1 or pXO2 produced negative results for the loci specific to these plasmids (Table). In addition, all 317 B. anthracis isolates from the 2001 outbreak were also positive for all three PCR targets (Table).

None of the 56 archived non–B. anthracis isolates, representing five other Bacillus species was positive for any of the three LRN PCR targets, including the Bacillus spp. isolate, Ba813_11, resulting in 100% specificity (95% CI 94% to 100%). Results were also negative for 88 clinical and environmental isolates, which were determined by standard microbiologic methods not to be B. anthracis (specificity 100%, 95% CI 96% to 100%).

The limit of detection on the LightCycler, Smart Cycler, and ABI Prism 7700 instruments, as determined by using DNA extracted from vegetative cells of the Sterne and Pasteur reference strains, was 1 pg DNA (approximately 167 cells based on a 5.5 Mbp genome size). Five to 10 spores could be detected on the ABI Prism 7700 instrument for the Ames (2000031656), Pakistan-sheep (2000031648), French-bovine (2000031651), and Sterne (2000031075) strains of B. anthracis.

The recent bioterrorism-associated anthrax outbreak demonstrated the need for sensitive, specific, and rapid methods for diagnosis and confirmation of anthrax, both for identification of suspect B. anthracis isolates and direct detection of B. anthracis DNA in clinical specimens. When tested on >500 strains, representing B. anthracis and five other Bacillus species, the LRN PCR exhibited 100% sensitivity and specificity.

To date, designing PCR assays for identification of B. anthracis has primarily focused on genes located on the plasmids (1–4). Patra et al. used a PCR that targeted two chromosomal loci, vrrA and Ba813, and found numerous environmental Bacillus isolates other than B. anthracis that were positive for both Ba813 and vrrA (11). While assays focusing on plasmid targets allow for a high level of specificity, a specific chromosomal target for detection of avirulent and plasmid-cured B. anthracis strains is needed. Thus, the LRN PCR includes a chromosomal target in addition to targets on each of the two virulence plasmids, pXO1 and pXO2.

Closely related B. cereus and B. thuringiensis, notorious for generating false-positive results using assays designed to be specific for B. anthracis (11,13), were consistently negative in this real-time PCR assay. B. anthracis, B. cereus, and B. thuringiensis are so closely related that their distinction as separate species is frequently questioned based on DNA-DNA hybridization studies, multiple-locus enzyme electrophoresis, and 16S rRNA sequence similarity (19–21). We have selected non–B. anthracis isolates that were primarily of clinical as opposed to environmental origin. B. cereus and B. thuringiensis clinical isolates are even more closely related to B. anthracis than their environmental counterparts (19,22), and they are more likely to cause false-positive results. We also tested the Bacillus spp. isolate that caused the one false-positive result in the Qi et al. report (13). Despite all of these challenges, all three targets of this real-time PCR assay have demonstrated 100% specificity and sensitivity in identification of B. anthracis when tested against our panel of Bacillus spp. strains and in identification of 317 outbreak-associated B. anthracis isolates. This LRN PCR is currently the only real-time PCR assay that detects both plasmid and chromosomal targets with 100% specificity and sensitivity. In addition, real-time PCR assays using fluorescent probes provide great sensitivity; this assay was able to detect 1 pg of purified DNA from vegetative cells (equivalent to 167 cells) or directly detect 5–10 spores.

The high level of sensitivity and specificity of the LRN PCR assay can be attributed to several factors. An extensive panel of DNA samples (non-Bacillus gram-positive bacterial species, gram-negative bacterial species, and human, vertebrate, and invertebrate DNA) were tested (data not shown). Having more than a single target decreases the rate of both false-negative and false-positive results, as they are not dependent on a single locus. The use of multiple targets also decreases the risk of false-positive results from contamination because each target is amplified as a separate PCR reaction. Finally, 5´ nuclease assays makes use of a fluorescent oligonucleotide probe, in addition to the forward and reverse primers, that allows for a lower limit of detection compared to conventional PCR, eliminates the need for post-PCR processing, and increases specificity (23,24).

The LRN PCR was shown to be important for use on environmental and clinical specimens during the 2001 bioterrorism-associated anthrax outbreak. A supplement covering the use of this assay on these specimens can be seen online (available from http://www.cdc.gov/ncidod/EID/vol8no10/02-0393sup.htm). The LRN PCR assay is widely available at over 200 laboratories in several countries and all 50 states of the United States through the Laboratory Response Network. The system is designed to be accessed through the State Department of Health.
