# Correction, Vol. 8, No. 10

**DOI:** 10.3201/eid0904.ER0203

**Published:** 2003-04

In the letter to the editor, Evaluation and Validation of a Real-Time Polymerase Chain Reaction Assay for Rapid Identification of Bacillus anthracis, the author list should read as follows:

Alex R. Hoffmaster, Richard F. Meyer, Michael P. Bowen, Chung K. Marston, Robbin S. Weyant, Kathy Thurman, Sharon L. Messenger, Erin E. Minor, Jonas M. Winchell, Max V. Rasmussen, Bruce R. Newton, J. Todd Parker, William E. Morrill, Nancy McKinney, Gwen A. Barnett, James J. Sejvar, John A. Jernigan, Bradley A. Perkins, and Tanja Popovic.

The corrected article appears online at http://www.cdc.gov/ncidod/eid/vol8no10/02-0393.htm

We regret any confusion these errors may have caused.

